# Bioassay-guided evaluation of *Dioscorea villosa* – an acute and subchronic toxicity, antinociceptive and anti-inflammatory approach

**DOI:** 10.1186/1472-6882-13-195

**Published:** 2013-07-28

**Authors:** Claudio Moreira Lima, Adriana Karla Lima, Marcelia G Dória Melo, Mairim Russo Serafini, Dênisson Lima Oliveira, Enrik Barbosa de Almeida, Rosana Souza Siqueira Barreto, Paulo Cesar de Lima Nogueira, Valéria Regina de Souza Moraes, Édica Ramone Andrade Oliveira, Ricardo Luiz Cavalcanti de Albuquerque Jr, Lucindo J Quintans-Júnior, Adriano Antunes Souza Araújo

**Affiliations:** 1 Department of Physiology, Federal University of Sergipe-UFS, São Cristóvão-SE CEP 49000-100, Brazil; 2 Laboratory of Morphology and Structural Biology Science and Technology Institute -ITP, Aracaju, SE CEP 49000-000, Brazil; 3 Tiradentes University, Aracaju, SE CEP 49000-000, Brazil; 4 Department of Chemistry, Federal University of Sergipe, São Cristóvão, SE 49100-000, Brazil

**Keywords:** *Dioscorea villosa*, Toxicity, Antinociceptive effect, Anti-inflammatory effect

## Abstract

**Background:**

*Dioscorea villosa* (DV) has been used in Brazil as an alternative medicine to attenuate menopause symptoms, as well as for the treatment of joint pain and rheumatoid arthritis. In spite of the popular use of DV for the treatment of various disorders, there are limited scientific data regarding safety aspects of this herb. In this regard, we carried out to evaluated both antinociceptive and anti-inflammatory activities in experimental models and assess the toxic effects of the acute (single dose) and subchronic (30 days) oral administration of dry extract of *Dioscorea villosa* in rodents.

**Methods:**

The LC analyses were performed to assess the presence of the diosgenin in samples of DV. The antinociceptive study of DV was performed using models of acetic acid-induced writhing and formalin-induced pain in mice. The anti-inflammatory study was accomplished by leukocyte migration to the peritoneal cavity. A dry extract of DV was tested at doses of 100, 200 and 400 mg/kg (*per os* or p.o.). The toxicological properties of the dry extract were evaluated by toxicity assays of acute (5 g/kg, single dose) and subchronic (1 g/kg/day, 30 days) treatment. Haematological, biochemical, and histopathological parameters were studied. The results are expressed as mean ± S.D., and statistical analysis of the data were performed with the Student’s *t*-test or one-way analysis of variance (ANOVA) followed by Tukey’s test. In all cases differences were considered significant if p < 0.05.

**Results:**

HPLC-DAD analysis of the extract from DV revealed the presence of diosgenin as the major compound. Doses of 200 and 400 mg⁄kg significantly reduced the amount of acetic acid-induced writhing in relation to the vehicle (p < 0.0001). In the first phase, using the formalin-induced neurogenic pain test, only the 400 mg/kg dose of DV showed significant inhibition of neurogenic pain (p < 0.001). In the second phase, 200 and 400 mg/kg of DV showed significant inhibition of inflammatory pain (p < 0.0001). Significant inhibition of leukocyte migration was observed with doses of 100 (p < 0.001), 200 (p < 0.01) and 400 mg/kg (p < 0.01). Haematological, biochemical and histopathological data obtained in both acute and subchronic toxicological assays revealed only unremarkable changes, which are unlikely to indicate DV toxicity with oral administration.

**Conclusion:**

We found that DV possesses antinociceptive and anti-inflammatory properties in rodent models. In addition, no acute or subchronic toxicity was evident when the herbal extract was administered orally. These results supporting the folkloric usage of the plant to treat various inflammatory diseases.

## Background

Ethnopharmacology is the cross-cultural scientific study of ethnic groups and their common knowledge about the use of plants in health care. Such ethnic knowledge of the herbal has been largely used to provide support for the production of medicines in order to improve the life quality of the populations [1,2]. In Brazil, the use of products derived from medicinal plants is regulated by the National Agency of Sanitary Surveillance through the law of herbal medicines (RDC n° 14 of 31/03/2010), which aims to ensure the quality of herbal products [3]. Several factors have encouraged the increasing use of herbal medicine in Brazil, such as biodiversity, variety of pharmacological activities, low cost to patients, reduced side effects and toxicity as well as a large acceptance of products based on medicinal plants. However, despite the advantages associated with natural products, it has been reported that their inadvertent overuse can damage health [1,4-6].

There is a strong trend in the use of natural substances with therapeutic activity, particularly in diseases in which clinically used synthetic drugs, such as analgesic, anti-inflammatory and hormonal drugs have several complications and side effects. These adverse reactions have propelled the search for safer alternative therapies in the last decades, such as the use of herbal products [1,2,7].

*Dioscorea villosa* (DV), commonly known as “yam”, is a medicinal woodland herb native from the temperate forests of eastern North America. The chemical composition of DV includes protodioscin, methylprotodioscin, dioscine, prosapogenin, epiafzelechin glucopyranoside, saponin glycosides, steroidal saponin, diosgenin, alkaloids, tannins and phytoestrogen [8-12]. In addition, the rhizomes and roots of DV have been popularly used as a non-conventional treatment of the symptoms of menopause [13,14], rheumatoid arthritis and hypoprogesteronaemia [8,11].

The safety, relatively low cost and easy availability of natural active products make them relevant models for the synthesis of more selective and powerful drugs. Although a considerable number of analgesic and anti-inflammatory drugs are currently available for clinical usage, the wide range of side-effects has led to the search for new compounds based on safer herbal medicine [6,15-17].

Despite the popular use of DV for the treatment of various disorders, there is limited scientific data available regarding the safety aspects of this herb, and there are no documented toxicological and pharmacologic studies asserting the safety index of herbal preparations. Therefore, the current study aimed to assess the toxicological (acute and subchronic) and pharmacological (anti-inflammatory and antinociceptive) properties of the dry extract of *Dioscorea villosa* in a rodent model.

## Methods

### Plant material

The dry extract of the *Dioscorea villosa* (DV) root was obtained from Shaanxi Meih Biochemics Co., Ltd. (Lot number MH-06DI-090609), China (imported by Galena Química e Farmacêutica Ltda company, São Paulo, SP, Brazil).

### Reagents

Diosgenin (≥93%) was purchased from Sigma-Aldrich (St. Louis, MO, USA), LC-grade acetonitrile (JT Baker, Philipsburg, PA, USA) was used for LC analysis and methanol (Tedia, Fairfield, OH, USA) was used for sample preparation. Deionized water was purified by a Milli-Q system (Millipore, São Paulo, SP, Brazil). All the solvents were filtered through nylon 0.45 μm membranes (MFS) and degassed by ultrasonic bath before use.

### Phytochemical studies

The LC analyses were performed on a Shimadzu liquid chromatographic (Kyoto, Japan) Prominence system, equipped with a degasser DGU-20A5 Model, SIL-10A autosampler, two high pressure pumps LC-20AT, a SPD-M10Avp photodiode array detector (DAD) and a CBM 20A interface. Data collection was performed using LC Solution software. Analysis was carried out on the analytical RP-18A Synergi® C_18_ column (5 μm, 250 × 4.6 mm i.d., Phenomenex, Torrance, CA, USA) with a RP-18A C_18_ guard column (4 μm, 4 × 3 mm, Phenomenex, Torrance, CA, USA) using a gradient elution at 1.0 mL/min with a mobile phase consisting of water (A) and acetonitrile (B). Initially, an exploratory gradient from 5 to 100% (B) in 60 min was run as suggested by Snyder et al. (1997) [18]. After optimization, the gradient elution condition used was: 30-40% (B) in 5 min, 40-68% (B) in 18 min, 68-100% (B) in 21 min, maintained in 100% (B) for 10 min. The return to initial chromatographic conditions (100-30% B) was doing in 10 min, followed by column conditioning for 10 min. The photodiode array detector was set at 205 nm for acquiring the chromatograms.

Samples of extract and diosgenin were dissolved in methanol:water (1:1 v/v) and methanol, respectively, at concentrations of 1.0 mg/mL each, and submitted to filtration through a cellulose membrane (pore diameter of 0.45 μm). Identification was based on co-injection of the standard compound and comparisons of absorption spectra.

### Animals

Male and female Wistar rats (150-200 g) and male Swiss mice (24-30 g) were obtained from the Tiradentes University (Sergipe, Brazil). The animals were kept at conventional temperature conditions (20 ± 1°C) and lodged in polypropylene cages with food and water available *ad libitum* and a 12 h light:dark cycle (light from 6 am to 6 pm). Experimental protocols were approved by the Ethical Committee in Animal Care of the Tiradentes University (CEPA/UNIT #110310R) and all procedures were carried out in accordance with Animal Care. The experiments were performed between 7 am and 5 pm.

### Antinociceptive study

#### Acetic acid-induced writhing

This study was performed according to Broadbear et al. (1994) [19] with alterations [6,20-22]. Mice (n = 6, per group) were injected intraperitoneally (i.p.) with 0.85% acetic acid at a dose of 10 ml/ kg. One hour before the acetic acid injection, the mice were pretreated orally (*per os* or p.o.) with DV (100, 200 and 400 mg/kg, *per os* or p.o.), morphine (MOR, 3 mg/kg, i.p.) or vehicle (0.9% saline with two drops of tween 80, the solvent for DV, Control group). Subsequently, writhing was counted for 15 min after a latency period of 5 min.

#### Formalin-induced pain

The procedure described by Hunskaar and Hole (1987) [23] was used with slight modifications [6,20-22]. Nociception was induced by injecting 20 μl of 1% formalin in distilled water in the subplantar region of the right hind paw. Mice (n = 6 per group) were given DV (100, 200 and 400 mg/kg) and vehicle (saline + two drops of tween 80) (*per os* or p.o.) 1,0 h prior to formalin injection. The acetylsalicylic acid (ASA, 200 mg/kg) and morphine (MOR, 3 mg/kg) were administered i.p. 0.5 hr before formalin injection. These mice were individually placed in a transparent acrylic glass cage (25 cm × 15 cm × 15 cm) observation chamber. The amount of time spent licking the injected paw was indicative of pain. After injection of formalin, licking time was recorded from 0-5 min (first phase) and 15–30 min (second phase), representing neurogenic and inflammatory pain responses, respectively.

### Anti-inflammatory study

Leukocyte migration to the peritoneal cavity: Leukocyte migration was induced by injection of carrageenan (500 μg/cavity, i.p., 500 μL) into the peritoneal cavity of mice 1 h after the administration of DV (100, 200 and 400 mg/kg, *per os* or p.o.), vehicle (saline 0.9% with two drops of tween 80) or dexamethasone (2 mg/kg, s.c., n = 6) using a modification of the technique previously described by Bastos et al. (2007) [24]. Mice were euthanized by cervical dislocation 4 h after carrageenan injection. Shortly afterward, phosphate buffered saline (PBS) containing EDTA (1 mM, i.p., 10 mL) was injected. Fluid was collected immediately using a brief massage and centrifuged (2000 rpm, 5 min) at room temperature. The supernatant was removed and 1 mL of PBS was introduced to the precipitate. A 10 μL aliquot from this suspension was dissolved in 200 μL of Turk’s solution and the total number of cells was counted in a Neubauer chamber, under an optical microscope. The results were expressed as leukocyte number per mL. The percentage of leukocyte inhibition was calculated as (1 – T/C) × 100, where T represents the leukocyte count of the treated group and C represents the leukocyte count of the control group [25].

### Toxicological studies

The following methods were performed according to the Organization for Economic Cooperation and Development test guidelines with slight modifications (OECD). The number of animals of this study was defined in compliance with Brazilian regulations for *in vivo* toxicological assays [26].

#### Subchronic toxicity study

Toxicological assays were performed with 20 male and 20 female rats distributed into 4 groups of 10 animals (experimental and control groups of male and female rats). For the subchronic study, the experimental groups received daily doses of DV (1 g/kg, *per os* or p.o.) dispersed in water (vehicle) over a period of 30 days. The product was stirred before being administered. The control groups received water only.

At the end of the period of administration, the animals were fasted for 12 h and then anesthetized with Ketamine (70 mg/kg) and Xylazine (12 mg/kg). Blood (3-5 ml) was collected by cardiac puncture. Subsequently, the animals were euthanized and a detailed study of the gross and microscopic features of the internal organs was performed, as well as haematological and biochemical analyses of the blood. The shape, size, texture, consistency and colour of the internal organs (lungs, heart, liver, stomach, pancreas, uterus/ovaries, brain and kidneys) were macroscopically observed for any sign of gross changes. The organs were then collected, weighed and preserved in 10% phosphate buffered formalin solution for subsequent histological procedures [4,22,26].

#### Acute toxicity study

Toxicological assays were performed with 12 male and 12 female rats, which were distributed into 4 groups of 6 animals (experimental and control groups of male and female rats). The animals received a maximum dose of 5 g/kg by oral administration (gavage). The experimental groups received DV (5 g/kg) dispersed in water (vehicle), whereas the control groups received water only. The product was stirred before being administered [26].

Specific behaviours (sedation, reduced ambulation, response to touch, analgesia and defecation) were observed and graded for 1, 2, 3 and 4 h after gavage. Finally, the animals were monitored daily for 14 days to observe the absence of lethality. At the end of the period of administration, the animals were fasted for 12 h, and then anesthetized with Ketamine (70 mg/kg) and Xylazine (12 mg/kg). Blood was collected by cardiac puncture. Subsequently, the animals were sacrificed and a detailed study of the gross and microscopic features of the internal organs was performed, as well as haematological and biochemical analyses of the blood. The position, shape, size, texture, consistency, and colour of the internal organs (lungs, heart, liver, stomach, pancreas, uterus/ovaries, brain and kidneys) were observed macroscopically, looking for any signs of gross changes. The organs were then collected, weighed and preserved in 10% phosphate buffered formalin solution for subsequent histological procedures [4,22,26].

### Parameters

#### Haematological analysis

Blood samples were collected into EDTA tubes. Measurements of erythrocytes, haemoglobin, haematocrit, leukocytes, neutrophils, lymphocytes, eosinophils, monocytes, basophils, VCM, HCM, CHCM, and platelets were determined using Sysmex Xs1000i automated equipment - Japan, according to the method described by Morris and Davey (1999) [27].

#### Biochemical parameters

Serum was separated from non-heparinised blood and assayed for serum urea, creatinine, total protein, globulin, albumin, aspartate aminotransferase (AST), alanine aminotransferase (ALT), total bilirubin (TBil), direct bilirubin (D.Bil), indirect bilirubin (Bil.IND), alkaline phosphatase (ALP), sodium (Na^+^), potassium (K^+^), cholesterol (CHOL), triglycerides (TRIG), glucose (GLUC) and uric acid. Biochemical parameters were determined by ARCHITECT C8000 automated equipment (Abbott, USA).

#### Tissue analysis

Formalin-fixed samples of the internal organs were dehydrated, diaphonized and embedded in paraffin according to protocols for routine histological procedures. Five micrometre thick sections of the paraffin-embedded tissues were obtained and stained with haematoxylin-eosin. Morphological analysis of the histological sections was performed by light microscopy following a closed numerical protocol so that the pathologist was not aware of what group was being evaluated until the end of the experiment.

### Statistical analysis

Student’s *t*-test (GraphPad Prism 5.01 computer program) was employed for statistical analysis of the results. The data obtained from the antinociceptive study were evaluated by one-way analysis of variance ANOVA followed by Tukey’s tests. The percentage inhibition by antinociceptive agent was determined using the following equation: (Eq. 1) % Inhibition = 100 × (control-experiment)/control. Statistical evaluation of the consumption of food and water was carried out using the differentiation factor (f1) and similarity factor (f2) methods [28]. All values were expressed as the back transformed mean ± S.D. or SEM. Differences below the probability level of 0.05 were considered statistically significant.

## Results and discussion

### Phytochemical studies

**Figure 1:**
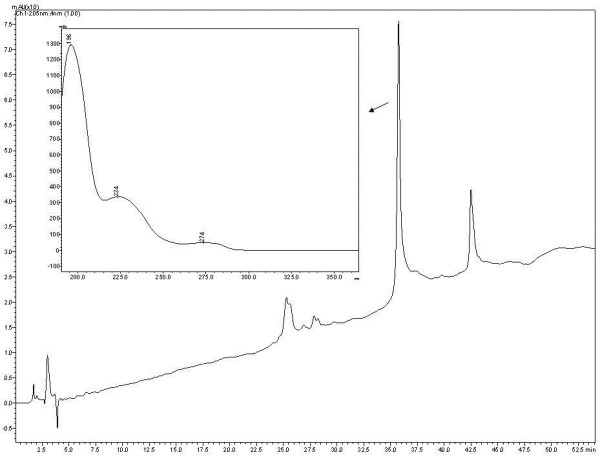
**HPLC-DAD chromatogram at 205 nm of the extract from *Dioscorea villosa.***

Qualitative analysis of the extract from DV was performed by HPLC. Figure 1 shows the chromatographic profile of this extract presenting good separation and resolution of peaks. The diosgenin was characterized by retention time relative to external standard. HPLC-DAD analysis of the extract from DV revealed the presence of diosgenin as the major compound.

### Antinociceptive studies

#### Acetic acid-induced writhing

**Figure 2:**
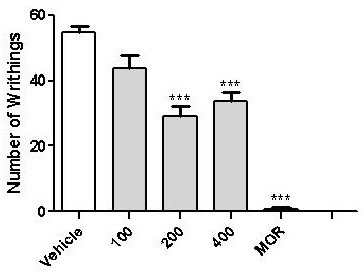
**Effects of *Dioscorea villosa *(DV) on the acetic acid-induced writhing test in mice.** Vehicle (control, *per os* or p.o.), morphine (MOR 3 mg/Kg, i.p) and DV (100, 200 and 400 mg⁄kg, *per os* or p.o.) were administered 1,0 hr before acetic acid injection. Each column represents mean ± S.E.M. (n = 6). ***p < 0.0001 versus control (ANOVA followed by Tukey’s test).

The acetic acid-induced writhing is a standard, simple and sensitive test for measuring analgesia induced by both opioids and peripherally acting analgesics [22,29]. According to Duarte et al. (1988) [30], the nociceptive mechanism involves the processing or release of arachidonic acid metabolites via cyclooxygenase (COX) and prostaglandin biosynthesis during abdominal writhing induced by acetic acid. In this study DV, in higher doses, significantly reduced writhing and stretching induced by i.p. administration of acetic acid solution (0.85% acetic acid). Significant protective effects were observed in Figure 2, through the inhibition (%) of nociceptive behaviour by 200 mg/kg (46.52%) and 400 mg/kg (38.46%) (p < 0.0001) doses of DV. MOR (3 mg/kg) had 98.17% (p < 0.0001) inhibition. These effects are probably related to the inhibition of PGE2 and PGF2α levels in the peritoneal fluid [31] and with the inhibited release of substance P and other inflammatory molecules such as serotonin, histamine, bradykinin, and prostaglandins [22,32].

#### Formalin-induced pain

**Figure 3:**
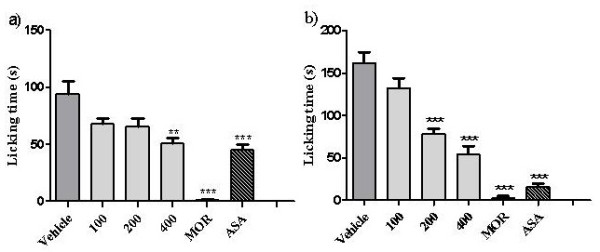
**Effects of *Dioscorea villosa *(DV) on the formalin-induced nociception in mice (first phase – Figure**2**a and second phase Figure**2**b).** Vehicle (control) and DV (100, 200 and 400 mg⁄kg) were administered *per os* or p.o. 1,0 hr before formalin injection. The acetylsali-cylic acid (ASA) or morphine (MOR) were administered i.p. 0.5 hr before formalin injection. Each column represents mean ± S.E.M. (n = 6). **p < 0.001 or ***p < 0.0001 versus control (ANOVA followed by Tukey’s test).

As shown by Tjølsen et al. (1992) [32], the formalin test involves continuous pain generated by injured tissue, and is a very useful method, not only for assessing antinociceptive drugs, but also to help elucidate the mechanism of action. The neurogenic phase is probably a direct result of C-fibre activation due to the peripheral stimulus in the paw and reflects centrally mediated pain with the release of substance P. The late phase appears to be dependent on the combination of inflammation in the peripheral tissue with the release of histamine, serotonin, bradykinin and prostaglandins [33-35]. *Dioscorea villosa* had analgesic effects in both the first and second phase of formalin-induced pain (p < 0.0001). In the first phase (Figure 3a) neurogenic-induced pain not was blocked at 100 (27.29% inhibition) or 200 mg/kg (p > 0.05) (30.06% inhibition), only the 400 mg/kg (46.06% inhibition) dose significantly blocked neurogenic pain. ASA (200 mg/kg; 52.2% inhibition) and MOR (98.76% inhibition) were significantly active in first phase (p < 0.05). This result shows the dose of 400 mg/kg is analgesic. In the second phase, representing inflammatory pain, DV significantly reduced licking time compared to the control group at 200 (51.36% inhibition) and 400 mg/kg (66.7% inhibition) (Figure 3b). Thus, higher doses of the extract were able to block both phases of the formalin response, but the effect was more prominent in the second phase.

Together these results support the hypothesis that DV participates in the inhibition of prostaglandin synthesis or the inhibition of proinflammatory cytokines (such as TNF-α) and suggests the partial involvement of analgesic central pathways.

### Anti-inflammatory studies

**Figure 4:**
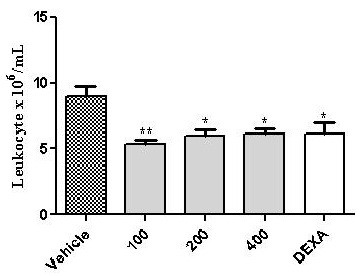
**Effect of *Dioscorea villosa *(DV) on leukocyte migration into the peritoneal cavity induced by carrageenan in mice.** Groups of rats were pre-treated with vehicle (control group, 10 mL/Kg, *per os* or p.o.), dexamethasone (Dexa, 2 mg/kg, s.c.), or DV in concentrations of 100, 200 and 400 mg/kg (*per os* or p.o.) 60 min before carrageenan (500 μg/cavity, 500 μL, i.p.)-induced peritonitis. Cell counts were performed at the time 4 h after the injection of carrageenan. Value represents the mean ± S.E.M. *p < 0.05 and **P < 0.001 when compared to control group. ANOVA followed by Tukey test (n = 6, per group).

The mechanism involved in the genesis of the carrageenan-induced oedema can cause the release of prostaglandins and kinins, as well as other substances such as prostaglandins, histamine, serotonin and leukotrienes. This type of oedema is dependent on the mobilisation of neutrophils [36-40]. The anti-inflammatory effect of DV can be observed from its inhibitory action on carrageenan-induced leukocyte migration to the peritoneal cavity 4 h after stimulus with all doses (p = 0.0027) compared to the control group, as shown in Figure 4. Significant inhibition effects of 40.41, 32.96 and 31.66% were observed for the 100, 200 and 400 mg/kg doses of DV*,* respectively. These results confirm the therapeutic activity of the plant when compared with vehicle, which did not exhibit activity. An effect of the drug dexamethasone (2 mg/kg, s.c.) on inhibited leukocyte migration was observed (31.75%), compared with the control group. As described by Loram et al., (2007) [41], inflammation induced by carrageenan involves cell migration, plasma exudation and production of mediators, such as interleukin (IL)-1β, IL-6 and tumour necrosis factor-alpha (TNF-α), a cytokine-induced neutrophil. These mediators are able to recruit leukocytes in several experimental models [25]. The decreased leukocyte migration in the carrageenan test suggests that DV activity may inhibit the synthesis of many inflammatory mediators whose involvement in cell migration is well established. These results may be related to the presence of diosgenin, a steroidal saponin found in several plants including species of *Solanum* and *Dioscorea*[42]. Previous investigations have shown that diosgenin plays an important pharmacological role as an anti-inflammatory agent [43] by inhibiting the release of cytokines, such as TNF-α [42].

### Toxicological studies

#### Toxicity study

In the acute toxicity test, a 5 g/kg dose of DV did not cause the death of any rats during a 14-day observation period. In addition, no change in body weight was observed in the experimental groups (Table 1). Because reduction in body weight gain and internal organ weights are regarded as simple and sensitive index of toxicity [44-46], our data suggest that oral administration of DV is not toxic. Surprisingly, one treated female (from day 5 to day 11) and two control (day 11), as well as one treated male (days 12 and 13) and two control (from day 12 to day 15) showed soft faeces in experimental day. Although it has been previously demonstrated that the ingestion of large amounts of the tubers can cause diarrhea [47], the fact that animals of both control and treated groups exhibited such alterations suggests that these data are unlikely to be side effects associated with administration of the dry extract. We also verified that the water consumption was decreased in treated male rats, but not in females, in relation to the control groups. Because no other behavioural changes were noticed in this study, it is possible to assert that the increased water consumption was unlikely to be related to any toxic effect of the acute experimental treatment.

**Table 1:** **Body weight of Wistar rats obtained after acute oral treatment with *Dioscorea villosa *dry extract at a daily dose of 5 g/Kg (single dose) given by oral route**

		**Control**		**DV 5000 mg/kg**	
**Gender**	**Day**	**Body weight (g)**	**Increase (%)**	**Body weight (g)**	**Increase (%)**
Male	0	332.14 ± 4.0	0.00	282.00 ± 5.2	0.00
	7	338.00 ± 5.5	1.76	284.00 ± 5.0	0.71
	14	345.43 ± 4.9	4.00	291.10 ± 5.6	3.24
					
Female	0	212.90 ± 4.6	0.00	195.50 ± 5.8	0.00
	07	214.00 ± 5.2	0.54	198.90 ± 5.3	1.73
	14	218.00 ± 5.2	2.42	203.10 ± 6.4	3.92

Values are expressed as mean ± S.E.M. (*n* = 07 animals/group).

On the other hand, in the subchronic assay (Table 2), animals of both genders treated with DV exhibited weight gain at rates significantly higher than control animals (p < 0.0001). These results might be related to the fact that diosgenin, one of the major chemical components of DV extract [9], is hypothesised to work as a biochemical precursor of progesterone and other corticoids [14]. Thus, the gain in body weight could be attributed to corticoid-associated fluid retention in response to the continuous exposure to the dry extract. This theory is supported by the absence of significant differences in food and water intake between treated and control groups.

**Table 2:** **Body weight of Wistar rats obtained after 30 consecutive days of treatment with *Dioscorea villosa *dry extract (DV) 1000 mg/kg given by oral route**

		**Control**		**DV 1000 mg/kg**	
**Gender**	**Day**	**Body weight (g)**	**Increase (%)**	**Body weight (g)**	**Increase (%)**
Male	0	352.6 ± 4.5	0.00	278.8 ± 5.1	0.00
	10	350.3 ± 4.4	−0.65	293.13 ± 4.9^***^	5.14
	20	362.2 ± 4.5	2.72	305.00 ± 3.9^***^	9.40
	30	364.0 ± 4.0	3.23	310.15 ± 4.2^***^	11.24
					
Female	0	195.1 ± 2.6	0.00	159.0 ± 3.5	0.00
	10	200.4 ± 3.5	2.72	176.0 ± 3.1^*^	10.69
	20	204.9 ± 3.1	5.02	189.7 ± 2.5^***^	19.30
	30	214.9 ± 4.2	10.15	196.4 ± 3.1^***^	23.50

Values are expressed as mean ± S.E.M. (*n* = 10 animals/group). * Significant difference from control group (*p < 0.05; **p < 0.01; ***p < 0.001 by ANOVA, followed by Tukey test).

As shown in Tables 3 and 4, there were distinct patterns of weight gain in some internal organs in male and female rats treated with DV in both acute and subchronic assays. We hypothesised that changes in the weight of these organs might be a result of specific activity of the chemical components of the extract in parenchymal cells, leading to mild hypertrophic or hyperplasic effects that are undetectable by gross or light microscopic examinations. In addition, because DV has been regarded as a phytohormone [8], differences in the number and distribution of hormone receptors in different genders might explain the fact that distinct organs were affected in male and female rats. However, further investigations are required in order to confirm this theory.

**Table 3:** **Changes in the organ weight of rats *Wistar *after acute oral treatment with *Dioscorea villosa *at a daily dose of 5 g/Kg (single dose)**

** Groups**				
**Organs**	**FC**	**FT**	**MC**	**MT**
Heart	0.4729 ± 0.015	0.4543 ± 0.011	0.4114 ± 0.010	0.3929 ± 0.015
Lungs	0.7343 ± 0.024	0.6700 ± 0.027	0.5529 ± 0.023	0.4786 ± 0.025
Liver	3.309 ± 0.1047	3.506 ± 0.1595	2.947 ± 0.080	2.960 ± 0.1238
Brain	0.7671 ± 0.022	0.8757 ± 0.022^**^	0.4914 ± 0.027	0.5414 ± 0.031
Kidneys	0.8071 ± 0.021	0.8271 ± 0.030	0.8329 ± 0.043	0.7671 ± 0.030
Stomach	0.8671 ± 0.032	0.7771 ± 0.015^*^	0.6571 ± 0.024	0.5686 ± 0.030^*^
Uterus/ovaries	0.4629 ± 0.089	0.7486 ± 0.097	-	-
Pancreas	0.4843 ± 0.092	0.5614 ± 0.060	0.3371 ± 0.097	0.3786 ± 0.043

*FC* control female, *FT* treated female, *MC* control male, *MT* treated male. Data are expressed as mean ± S.D., n = 6; * Significant difference from control group ( ^*^p < 0.05, ^**^p < 0.01 and ^***^p < 0.001) (test *t*).

**Table 4:** **Changes in the organ weight (g/%) of rats after subchronic oral treatment with *Dioscorea villosa *at a daily dose of 1000 mg/kg for 30 days**

** Groups**				
**Organs**	**FC**	**FT**	**MC**	**MT**
Heart	0.426 ± 0.055	0.447 ± 0.049	0.372 ± 0.021	0.414 ± 0.039
Lungs	0.631 ± 0.097	0.677 ± 0.067	0.523 ± 0.065	0.498 ± 0.093
Liver	3.084 ± 0.279	3.620 ± 0.518*	2.678 ± 0.083	2.993 ± 0.497
Brain	0.783 ± 0.055	0.892 ± 0.071**	0.511 ± 0.035	0.594 ± 0.059**
Kidneys	0.704 ± 0.040	0.843 ± 0.056***	0.756 ± 0.032	0.794 ± 0.041
Stomach	0.671 ± 0.065	0.760 ± 0.136	0.572 ± 0.059	0.507 ± 0.063*
Uterus/ovaries	0.363 ± 0.052	0.409 ± 0.068	-	-
Pancreas	0.528 ± 0.195	0.526 ± 0.057	0.553 ± 0.165	0.397 ± 0.090*

*FC* control female, *FT* treated female, *MC* control male, *MT* treated male. Data are expressed as mean ± S.D., n = 10; * Indicate statistical difference between the control and *Dioscorea villosa* group ^*^p < 0.05, ^**^p < 0.01 and ^***^p < 0.001 (test *t*).

#### Haematological analysis

Despite significant differences in some haematological parameters were verified in control and treated groups of both genders (Table 5 and 6), the data obtained in the acute and subchronic assays were within reference value ranges described in previous studies [21], suggesting that such alterations hold no clinical relevance [48]. Therefore, the slight alterations observed in this study concerning those parameters, particularly those in the control groups, might be associated to biological variation related to the nature and management of the animals in the different research vivaria.

**Table 5:** **Effect of *Dioscorea villosa *after oral administration (single dose-5 g/Kg) on hematologic parameters considering sex interaction in Wistar rats (*n*** **= 6)**

** Groups**						
**Parameters**	**FC**	**FT**	**R1**	**MC**	**MT**	**R2**
Leukocyte (×10^3^/mm^3^)	5.17 ± 0.853	7.18 ± 0.385	4.7 – 12.98 ^a^	7.03 ± 0.677	7.70 ± 1.220	5.7 – 13.9 ^a^
Erythrocytes (×10^6^/mm^3^)	7.89 ± 0.395	8.60 ± 0.135	7.3 – 8.64 ^a^	9.56 ± 0.249	9.66 ± 0.230	6.7 – 8.6^a^
Hemoglobin (g/dl)	13.43 ± 0.685	14.87 ± 0.225	13.2 – 15.1 ^a^	15.59 ± 0.417	15.91 ± 0.309	12.8 – 15.9 ^a^
Hematocrit (%)	42.93 ± 2.166	43.37 ± 0.569	39.1 – 48.5 ^a^	48.43 ± 1.206	45.60 ± 0.649	39.1 – 48.7 ^a^
VCM (fl)	54.40 ± 0.727	49.87 ± 0.201^***^	49.1 – 62.5 ^a^	50.70 ± 0.439	47.21 ± 0.472^***^	50 – 59 ^a^
HCM (pg)	17.00 ± 0.089	17.08 ± 0.065	16.6 – 18.9 ^a^	16.31 ± 0.112	16.5 ± 0.104	17.0 – 19.5 ^a^
CHCM (g/dl)	31.28 ± 0.433	34.27 ± 0.071^***^	29.9 – 34.9 ^a^	32.17 ± 0.191	34.91 ± 0.265^***^	30.5 – 35.3 ^a^
Platelets (×10^6^/mm^3^)	1070 ± 74.760	1246 ± 59.830	757 – 1476 ^a^	1198 ± 41.10	1141 ± 73.77	837 – 1455 ^a^
Neutrophil (%)	48.40 ± 7.259	36.97 ± 2.070	5.4 – 37.5 ^a^	45.84 ± 1.335	48.80 ± 3.384	17.1 – 47.9 ^a^
Lymphocyte (%)	47.46 ± 5.432	57.73 ± 2.464	57.9 – 90.0 ^a^	48.26 ± 1.716	46.09 ± 2.773	48.7 – 78.1 ^a^
Monocyte (%)	2.857 ± 0.739	3.43 ± 0.814	0.6 – 7.9 ^a^	3.47 ± 0.201	3.63 ± 0.432	1.0 – 6.5 ^a^
Eosinophil (%)	1.60 ± 0.323	1.30 ± 0.206	0.2 – 4.5 ^a^	0.16 ± 0.031	0.08 ± 0.016	0.3 – 4.0 ^a^

*FC* (control female), *FT* (treated female), *MC* (control male), *MT* (treated male). N = 06. Data are expressed as mean ± S.D. ^*^ Indicate statistical difference between the control and treated group ^*^p < 0.05, ^**^p < 0.01 and ^***^p < 0.001 (test *t*). *R1* (reference female); *R2* (reference male); ^a^ Melo (2012).

**Table 6:** **Effect of *Dioscorea villosa *after oral administration (30 days) on hematologic parameters interaction in Wistar rats (*n*** **= 10)**

**Parameters**	**FC**	**FT**	**R1 Female**	**MC**	**MT**	**R1 Male**
Leukocyte (×10^3^/mm^3^)	5.28 ± 0.896	7.16 ± 1.604	4.7 – 12.98^a^	8.20 ± 1.959	8.37 ± 1.376	5.7 – 13.9 ^a^
Erythrocytes (×10^6^/mm^3^)	8.15 ± 0.262	8.76 ± 0.421	7.3 – 8.64 ^a^	9.40 ± 0.339	9.62 ± 0.303	6.7 – 8.6 ^a^
Hemoglobin (g/dl)	14.38 ± 0.473	15.17 ± 0.566	13.2 – 15.1 ^a^	15.65 ± 0.602	16.33 ± 0.409*	12.8 – 15.9 ^a^
Hematocrit (%)	40.34 ± 1.571	42.11 ± 1.515	39.1 – 48.5 ^a^	42.53 ± 1.497	44.50 ± 0.818**	39.1 – 48.7 ^a^
VCM (fl)	49.49 ± 0.737	48.27 ± 1.250	49.1 – 62.5 ^a^	45.24 ± 0.964	46.33 ± 1.029*	50.0 – 59.0 ^a^
HCM (pg)	17.63 ± 0.149	17.28 ± 0.405	16.6 – 18.9 ^a^	16.64 ± 0.236	17.0 ± 0.252**	17.0 – 19.5^a^
CHCM (g/dl)	35.65 ± 0.299	35.82 ± 0.323	29.9 – 34.9 ^a^	36.80 ± 0.573	36.70 ± 0.472	30.5 – 35.3 ^a^
Platelets (×10^6^/mm^3^)	1115.30 ± 115.039	1356.30 ± 90.371***	757 – 1476 ^a^	1111.20 ± 135.711	1183.89 ± 66.196	837 – 1455 ^a^
Neutrophil (%)	36.64 ± 2.587	24.35 ± 4.461***	5.4 – 37.5 ^a^	41.80 ± 4.007	30.32 ± 5.052***	17.1 – 47.9 ^a^
Lymphocyte (%)	58.49 ± 3.222	71.59 ± 4.228***	57.9 – 90.0 ^a^	51.43 ± 3.969	64.18 ± 5.332***	48.7 – 78.1 ^a^
Monocyte (%)	4.42 ± 0.947	3.90 ± 0.587	0.6 – 7.9 ^a^	6.12 ± 1.353	4.70 ± 1.983	1.0 – 6.5 ^a^
Eosinophil (%)	0.45 ± 0.534	0.16 ± 0.101	0.2 – 4.5 ^a^	0.65 ± 0.701	0.23 ± 0.150	0.3 – 4.0 ^a^

*FC* (control female), *FT* (treated female), *MC* (control male), *MT* (treated male). N = 10. Data are expressed as mean ± S.D. ^*^ Indicate statistical difference between the control and treated group ^*^p < 0.05, ^**^p < 0.01 and ^***^p < 0.001 (test *t*). R1 (reference female); R2 (reference male); ^a^ Melo (2012).

#### Biochemical parameters

In the subchronic assay (Table 7), the increased biochemical level of ALP and D.BIL in treated rats (male and female) might be suggestive of cholestatic alterations secondary to changes in biliary flow [49]. In addition, increased levels of AST and ALT suggesting possible damage to the hepatocytes [49] were only observed in the treated females. However, the treated rats (both males and female) appeared to sustain the normal liver function, as there was no reduction in the production of protein or albumin when compared to the treated group. The values of urea and creatinine were not increased in the blood, indicating that renal function was not affected. The treated rats (male and female) showed hypernatraemia and hyperglycaemia compared with the control group (p < 0.05). Because DV has been reported as a corticoid precursor, these data are likely related to renal sodium retention and increased glycogenesis [50] promoted by saponins derived from the herbal dry extract.

**Table 7:** **Serum biochemical findings in rats treated with *Dioscorea villosa *at a daily dose of 1000 mg/kg for 30 days**

**Parameters**	**FC**	**FT**	**R1 Female**	**MC**	**MT**	**R1 Male**
Albumin (g/dL)	3.05 ± 0.284	3.33 ± 0.050**	2.6 – 3.4 ^a^	2.75 ± 0.085	3.10 ± 0.122***	2.7 – 3.2 ^a^
ALP (U/L)	35.90 ± 7.325	86.55 ± 15.428***	63 – 138 ^a^	48.20 ± 9.175	92.00 ± 17.356***	79 – 196 ^a^
TGP or ALT (U/L)	36.80 ± 3.765	46.11 ± 5.710***	26 – 60 ^a^	51.10 ± 3.814	53.55 ± 4.825	36 – 58 ^a^
TGO or AST (U/L)	68.90 ± 13.211	84.66 ± 15.851*	83 – 184 ^a^	99.30 ± 24.097	87.78 ± 18.633	81 – 180 ^a^
Creatinine (mg/dL)	0.53 ± 0.022	0.49 ± 0.022**	0.4 – 0.7 ^a^	0.45 ± 0.032	0.49 ± 0.027*	0.44 – 0.64 ^a^
GLUC (mg/dL)	128.70 ± 17.827	150.55 ± 9.302**	61 – 147 ^a^	139.70 ± 18.607	146.22 ± 12.163	79 – 144.0 ^a^
K^+^ (mmol/L)	4.07 ± 0.200	4.755 ± 0.557**	3.7 – 5.7 ^a^	4.86 ± 0.529	5.49 ± 1.128	4.2 – 6.4 ^a^
Na^+^ (mmol/L)	132.60 ± 0.699	138.55 ± 2.651***	132 - 146 ^a^	132.90 ± 1.197	140.66 ± 2.061***	135 - 144 ^a^
Total Protein (g/dL)	3.50 ± 0.271	6.16 ± 0.212***	6.1 – 7.4 ^a^	3.33 ± 0.125	5.99 ± 0.271	5.4 – 6.6 ^a^
TRIG (mg/dL)	29.20 ± 4.315	31.33 ± 6.633	39 – 130 ^a^	34.70 ± 3.683	30.00 ± 6.422	42 – 160 ^a^
Urea (mg/dL)	41.50 ± 4.972	40.00 ± 4.528	30 – 57 ^a^	36.80 ± 3.293	37.78 ± 2.438	30 – 42 ^a^
Uric acid (mg/dL)	1.83 ± 0.374	1.97 ± 0.630	1.2 – 2.5 ^a^	1.53 ± 0.327	1.60 ± 0.367	0.9 – 2.0 ^a^
Bil.IND (mg/dL)	0.15 ± 0.227	0.06 ± 0.055	0.01 – 0.01 ^a^	0.07 ± 0.005	0.07 ± 0.050	0.01 – 0.01 ^a^
D.Bil (mg/dL)	0.02 ± 0.006	0.10 ± 0.000***	0.01 – 0.03 ^a^	0.02 ± 0.005	0.10 ± 0.000	0.01 – 0.03 ^a^
TBil (mg/dL)	0.10 ± 0.000	0.15 ± 0.055*	0.07 – 0.09 ^a^	0.10 ± 0.000	0.16 ± 0.055	0.07 – 0.09 ^a^

*FC* (control female), *FT* (treated female), *MC* (control male), *MT* (treated male), N = 10. Data are expressed as mean ± S.D. ^*^ Indicate statistical difference between the control and treated group ^*^p < 0.05, ^**^p < 0.01 and ^***^p < 0.001 (test *t*). R1 (reference female); R2 (reference male) ^a^ Melo (2012).

As shown in Table 8, the biochemical results of the acute assay denoted isolated elevation of ALT in females and ALP in both sexes, which may be indicative of superficial injury of the hepatocyte [51]. The treated males presented a decrease in the production of total protein and albumin, suggesting impairment of liver function [52]. As in the subchronic test, only the females showed hyperglycaemia. On the other hand, both males and females treated rats had hypocholesterolaemia when compared to the control group. Because the treatment of menopause with synthetic hormones might lead to increases in cholesterol [53], our findings suggest that DV dry extract could be regarded as a safer phytomedicine to control climacteric symptoms without causing side effects on serum fat levels. Despite some significant differences in other biochemical parameters observed in this assay, they were within the reference values for the species studied. None of these significant differences were considered to be toxicologically relevant due to a lack of dose-response relationship, relatively low magnitude of change, absence of occurrence in both sexes, and/or the lack of important changes in related clinical parameters. Also, no abnormalities or further evidence of histopathological changes were seen in any of the control or treated rats of both sexes.

**Table 8:** **Serum biochemical findings in rats *Wistar *treated with *Dioscorea villosa *at a single dose (5 g/kg)**

**Parameters**	**FC**	**FT**	**R1 Female**	**MC**	**MT**	**R1 Male**
Albumin (g/dL)	3.06 ± 0.097	3.06 ± 0.030	2.6 – 3.4 ^a^	3.03 ± 0.112	2.73 ± 0.042^*^	2.7 – 3.2 ^a^
ALP (U/L)	48.00 ± 4.669	73.43 ± 5.622^**^	63 – 138 ^a^	80.00 ± 4.889	109.4 ± 6.575^**^	79 – 196 ^a^
TGP or ALT (U/L)	40.60 ± 3.614	51.57 ± 2.999^*^	26 – 60 ^a^	68.43 ± 7.234	62.43 ± 2.653	36 – 58 ^a^
TGO or AST (U/L)	106.80 ± 8.509	86.00 ± 5.499	83 – 184 ^a^	160.9 ± 15.33	121.1 ± 14.12	81 – 180 ^a^
Creatinine (mg/dL)	0.49 ± 0.007	0.5329 ± 0.015	0.4 – 0.7 ^a^	0.59 ± 0.019	0.51 ± 0.007^**^	0.44 – 0.64 ^a^
GLUC (mg/dL)	131.40 ± 7.593	161.7 ± 5.222^**^	61 - 147 ^a^	157.6 ± 7.030	172.7 ± 7.819	79 – 144 ^a^
K^+^ (mmol/L)	4.05 ± 0.193	4.06 ± 0.146	3.7 – 5.7 ^a^	5.814 ± 0.370	5.07 ± 0.185	4.2 – 6.4 ^a^
Na^+^ (mmol/L)	133.40 ± 0.812	134.10 ± 0.459	132 – 146 ^a^	135.6 ± 1.478	135.3 ± 0.356	135 – 144 ^a^
Total Protein (g/dL)	4.17 ± 0.063	4.27 ± 0.052	6.1 – 7.4 ^a^	4.614 ± 0.230	3.81 ± 0.103^**^	5.4 – 6.6 ^a^
TRIG (mg/dL)	32.75 ± 2.136	42.57 ± 3.683	39 – 130 ^a^	46.57 ± 3.077	54.57 ± 6.294	42 – 160 ^a^
Urea (mg/dL)	33.60 ± 1.503	37.14 ± 1.668	30 – 57 ^a^	47.43 ± 1.251	37.57 ± 1.232	30 – 42 ^a^
Uric acid (mg/dL)	1.65 ± 0.132	2.04 ± 0.169	1.2 – 2.5 ^a^	3.37 ± 0.377	2.41 ± 0.298	0.9 – 2.0 ^a^
Bil.IND (mg/dL)	0.08 ± 0.004	0.08 ± 0.003	0.01 – 0.01 ^a^	0.07 ± 0.011	0.08 ± 0.003	0.01 – 0.01 ^a^
D.Bil (mg/dL)	0.02 ± 0.004	0.02 ± 0.003	0.01 – 0.03 ^a^	0.03 ± 0.011	0.02 ± 0.003	0.01 – 0.03 ^a^
TBil (mg/dL)	0.10 ± 0.004	0.10 ± 0.003	0.07 – 0.09 ^a^	0.10 ± 0.010	0.10 ± 0.003	0.07 – 0.09 ^a^
CHOL (mg/dL)	60.60 ± 2.315	56.57 ± 2.599	55 – 79 ^a^	59.57 ± 3.191	50.14 ± 2.405	46 – 101 ^a^

*FC* (control female), *FT* (treated female), *MC* (control male), *MT* (treated male). N = 10. Data are expressed as mean ± S.D. ^*^ Indicate statistical difference between the control and treated group ^*^p < 0.05, ^**^p < 0.01 and ^***^p < 0.001 (test *t*). R1 (reference female); R2 (reference male); ^a^ Melo (2012).

#### Tissue analysis

Pathological examination of tissues indicated no detectable abnormality, either in weight or in appearance, between control and test groups. Moreover, the organs of both groups were unremarkable and comparable within each sex. The tissue analysed did not exhibit histological changes, except for the liver, which presented foci of intracellular accumulation of fatty droplets, consistent with microvesicular steatosis, in the acute and subchronic assays in both genders.

While microvesicular steatosis has usually been described in association with severe clinical cases of hepatic damages, previous finding suggest that it can be present without liver dysfunction or hepatotoxicity [54]. Therefore, it seems that further investigations are still required to clarify the mechanisms underlying the pathogenesis of microvesicular steatosis, particularly in order to better establish the clinical implications of this morphological hepatic change. In addition, alterations in biochemical measurements, indicative of cholestasis (subchronic test) and represented histologically as accumulation of bilirubin in the sinusoidal spaces, hepatocyte injury, and inflammation (accumulation of immune cells) were not confirmed by the histological analysis, suggesting that these supposed disturbances were not expressive enough to promote morphological changes in the hepatic tissue. These findings indicate that oral DV promotes no morphological changes in the vital organs of animals and suggest that this substance produces no toxicological effects.

## Conclusion

It can be concluded that DV is effective as an analgesic in two pain models, and provides expressive anti-inflammatory effect. The extract presented no acute and subchronic toxicity when administered by oral route. Further studies are currently in progress to clarify the precise mechanisms of action of DV on nociception and inflammation. However, the antinociceptive and anti-inflammatory actions demonstrated in *Dioscorea villosa* in this study support at least in part, the ethnomedicinal use of this plant.

## Abbreviations

DV: *Dioscorea villosa*; AST: Aspartate aminotransferase; ALT: Alanine aminotransferase; TBil: Total bilirubin; D.Bil: Direct bilirubin; Bil.IND: Indirect bilirubin; ALP: Alkaline phosphatise; Na+: Sodium; K+: Potassium; CHOL: Cholesterol; TRIG: Triglycerides; GLUC: Glucose.

## Competing interests

The authors declare that they have no competing interests.

## Authors’ contributions

CML was responsible for the conception and design, carried out all experiments, performed data analysis and drafted the manuscript. AKL and DLO were responsible for the carried the toxicological studies. MGDM participated in the biochemical analysis. MRS collaborated in data analysis. EBA was responsible for the tissue analysis. RSSB collaborated in anti-inflammatory studies. RLCAJr made contribution in the revised of the manuscript and tissue analysis. ERAO, VRSM and PCLN participated in the chromatographic analysis. LJQJr collaborated in monitoring pharmacological studies. AASA made contribution to conception and revised of the manuscript. All authors read and approved the final manuscript.

## Pre-publication history

The pre-publication history for this paper can be accessed here:

http://www.biomedcentral.com/1472-6882/13/195/prepub
